# A tilt after-effect for images of buildings: evidence of selectivity for the orientation of everyday scenes

**DOI:** 10.1098/rsos.160551

**Published:** 2016-11-23

**Authors:** Ahamed Miflah Hussain Ismail, Joshua A. Solomon, Miles Hansard, Isabelle Mareschal

**Affiliations:** 1 Department of Experimental Psychology, School of Biological and Chemical Sciences, Queen Mary University of London, London, UK; 2 School of Electronic Engineering and Computer Science, Queen Mary University of London, London, UK; 3 Centre for Applied Vision Research City, University of London, London, UK

**Keywords:** natural images, global orientation, tilt after-effect, spatially non-specific

## Abstract

The tilt after-effect (TAE) is thought to be a manifestation of gain control in mechanisms selective for spatial orientation in visual stimuli. It has been demonstrated with luminance-defined stripes, contrast-defined stripes, orientation-defined stripes and even with natural images. Of course, all images can be decomposed into a sum of stripes, so it should not be surprising to find a TAE when adapting and test images contain stripes that differ by 15° or so. We show this latter condition is not necessary for the TAE with natural images: adaptation to slightly tilted and vertically filtered houses produced a ‘repulsive’ bias in the perceived orientation of horizontally filtered houses. These results suggest gain control in mechanisms selective for spatial orientation in natural images.

## 1. Introduction

Gibson & Radner [1] demonstrated that adapting to a line tilted between 2.5° and 45° from vertical makes a vertical ‘test’ stimulus, presented in the same retinal location, appear tilted in a direction opposite to that of the adaptor. This repulsive effect on perceived orientation is known as the tilt after-effect (TAE). Most contemporary theories commonly attribute the TAE to suppression of responses in neurons tuned to the adaptor's orientation [2], either via fatigue of the adapted neurons [3] or lateral inhibition between neurons with similar orientation preferences [4,5], although other accounts have been proposed [6]. The TAE is a natural consequence of orientation-selective suppression, which effectively skews neural responses away from the adapting orientation.

Any repulsive after-effect can be considered as evidence for the existence of neural populations selectively encoding a specific stimulus feature. Consequently, after-effects have earned a reputation for being ‘the psychophysicist's micro-electrode’ [7]. Using after-effects, psychophysicists have inferred the existence of neural selectivity for such complex attributes as shape, glossiness and facial expression [8]. There is even an after-effect of adaptation to heavily masculine or feminine features [9]. However, it must be acknowledged that some of these after-effects might be the result of adaptation in ‘low-level’ visual mechanisms, tuned to stimulus values that have nothing to do with faces *per se*. For example, if adapting to a thick, masculine eyebrow suppresses a few neurons that prefer (low spatial frequency (SF)) shapes like that, then a subsequently viewed, androgynous eyebrow (with a slightly higher SF) will appear much thinner, making the face it is on appear more feminine. Thus, inferring neural mechanisms from perceptual after-effects is not always as straightforward as one might hope.

Inferring neural selectivity from psychophysics is complicated, not only because after-effects can reflect adaptation by low-level mechanisms, but also because many conventional measurements of appearance are susceptible to contamination from non-perceptual sources of bias (e.g. expectation effects and response biases; [10]). In this study, we minimize the influence of low-level adaptation by restricting adaptor and tests to different regions of the visual field and/or different regions of frequency space. We minimize the influence of non-perceptual sources of bias by adopting the recently developed, two-alternative, forced-choice (2AFC) comparison-of-comparisons paradigm, with roving pedestals [11,12].

The after-effect we have studied is the recently reported TAE for natural scenes [13]. Global scene orientation is important for a number of reasons. Firstly, perceived orientation of a scene provides information about the direction of gravity, which in turn informs self-orientation relative to gravity. This is particularly relevant when information provided by other sensory sources is discordant [14]. Secondly, judgements of subjective visual vertical are affected by the orientation of background scenes, which serve as a global frame of reference for perceptual judgements [15,16]. Finally, it has been reported that scene orientation affects how people deploy overt attention within a scene, where scene-centric directional asymmetries of eye movements always remain aligned with the orientation of the scene [17].

In Experiment 1, we confirm that the TAE for natural scenes can be obtained with different (and differently sized) adapting and test images, which are presented in a partially overlapping spatial configuration and share minimal SF components. In Experiment 2, the specific question we address is whether the TAE for natural scenes arises because of interactions between mechanisms selective for natural scenes, or whether it is simply a by-product of suppression between more lower level mechanisms, selective for spatial orientation in general. To disentangle these possibilities, we use orientation-filtered and phase-scrambled stimuli. Vertically filtered images are designed to have a negligible effect on the responsivity of low-level mechanisms tuned to near-horizontal orientations. Phase-scrambled stimuli are designed to have a similarly negligible effect on the responsivity of mechanisms selective for natural scenes.

## 2. Material and methods

### 2.1. Participants

A total of 23 observers (18–46 years of age), each having a unique two-character set of initials (figures 2 and 3), from Queen Mary University of London with normal or corrected-to-normal visual acuity took part in the experiments. The number of participants for each experimental condition was determined based on previous studies investigating higher level visual after-effects, which involved from 5 to 10 observers per condition [18–20].

### 2.2. Experimental set-up and apparatus

Observers were seated in a dark room and were instructed to keep their head upright and maintain the same distance from the screen throughout the experiment. Stimuli were presented on a 20^″^ Iiyama CRT monitor with a 1600 × 1200 screen resolution and a refresh rate of 60 Hz. The viewing distance was 57 cm, such that each pixel subtended 1.5 arcmin. A black mask with a circular aperture (diameter = 24.5°) was overlaid on the monitor to eliminate the use of monitor edges as cues to vertical or horizontal. Stimulus presentation and data collection used Matlab (Mathworks) and Psychtoolbox [21].

### 2.3. Stimuli

**Figure 1.**
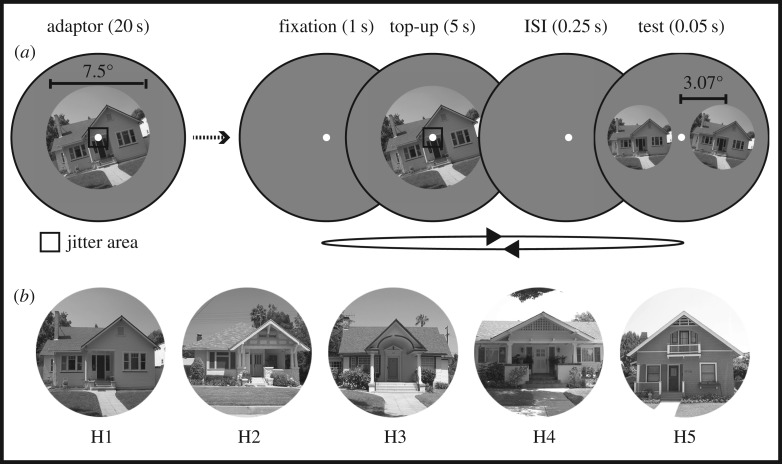
(*a*) Stimulus configuration and timeline of a sample trial from Experiment 1. (*b*) Five different house scenes used across the different conditions in the study.

Images of five different houses (figure 1*b*), in their frontal views, appearing to be at eye level from a standing position, were obtained from an archive of the Caltech Computational Vision Group (http://www.vision.caltech.edu/archive.html). We used images of houses because: (i) scene orientation of man-made scenes is judged with better discrimination precision than non-man-made scenes [16] and (ii) houses have a clear frontal facade and cover limited depth, resulting in minimal linear perspectives. The images were initially cropped to a square aspect ratio and then resized to 300 × 300 pixels using bicubic interpolation. Cropped images were converted to greyscale by independently weighting and summing the red, green and blue channels of the image according to the CIE procedure (0.299 × R + 0.587 × G + 0.114 × B). These images were presented as adaptors within a hard-edged circular aperture (diameter = 7.5°; figure 1*a*). The test images were resized to 75% of the adaptor's size and presented within a hard-edged window of diameter 5.7°.

Images of houses were tilted and, in some cases, filtered. Filtering was a 7-step procedure. In step 1, the mean greylevel of a tilted image was subtracted, creating a difference image with no DC component. In step 2, this difference image was multiplied with a two-dimensional, separable cosine window of the same size. In step 3, the windowed image was Fourier transformed (applying the cosine window before Fourier transformation helps to reduce wrap-around artefacts). In step 4, the transformed image was multiplied by one of the filters described below. In step 5, the product was inverse-Fourier transformed. In step 6, the image was scaled such that adaptors would have a root mean square (RMS) contrast of 0.10 and tests would have an RMS contrast of 0.18. Finally, in step 7, a greylevel of 0.50 was added to each image. This matched the greylevel of the screen background.

### 2.4. Procedure

Trials were blocked by condition (there were three conditions in Experiment 1 and two conditions in Experiment 2) and adaptor orientation: either –15° or +15°. By convention, we consider tilts clockwise (CW) from vertical to be negative and tilts counter-clockwise (CCW) from vertical to be positive. Each condition in Experiments 1 and 2 was also associated with a ‘baseline block’, in which no adaptor was shown.

The general procedure is outlined in figure 1*a*. Observers were instructed to fixate a centrally presented white circle (diameter = 0.2°) for the duration of each block. All blocks (except baseline blocks) began with an initial adaptation phase of 20 s. Following this, each test trial started with a ‘top-up’ adaptation phase of 5 s. During adaptation phases, the adaptor was jittered every 0.5 s by recentring it on a random pixel within a predefined jitter area of 0.25° × 0.25° surrounding fixation. Top-up adaptors were followed, after 0.25 s, by two test houses, presented immediately to the left and right of fixation, for 0.05 s. One of the test houses was the ‘pedestal’, with one of two fixed tilts: –3° or +3°. The other test was the ‘comparison’, with an offset added to the fixed tilt, randomly selected from the set {–15°, –12°, –9°, –6°, –3°, 0°, +3°, +6°, +9°, +12°, +15°}. Each combination of pedestal and comparison tilt was tested 10 times, resulting in 220 trials per block. The spatial positions (left and right of fixation) of the pedestal and comparison were randomized on every trial. Observers chose which of the two test houses appeared more upright, using keys ‘1’ (for left) and ‘2’ (for right). Observers were told that an upright house is how they would imagine it to appear, if they stood in front of it with their head held straight.

As is evident from figure 1*a*, there was a small amount of spatial overlap between the adaptor and tests. However, the overlapping parts of the images were not the same (e.g. the right half of the adaptor overlapped with the left half of one test) and were of different sizes to reduce retinotopic adaptation [22].

### 2.5. Methods specific to Experiment 1

**Figure 2.**
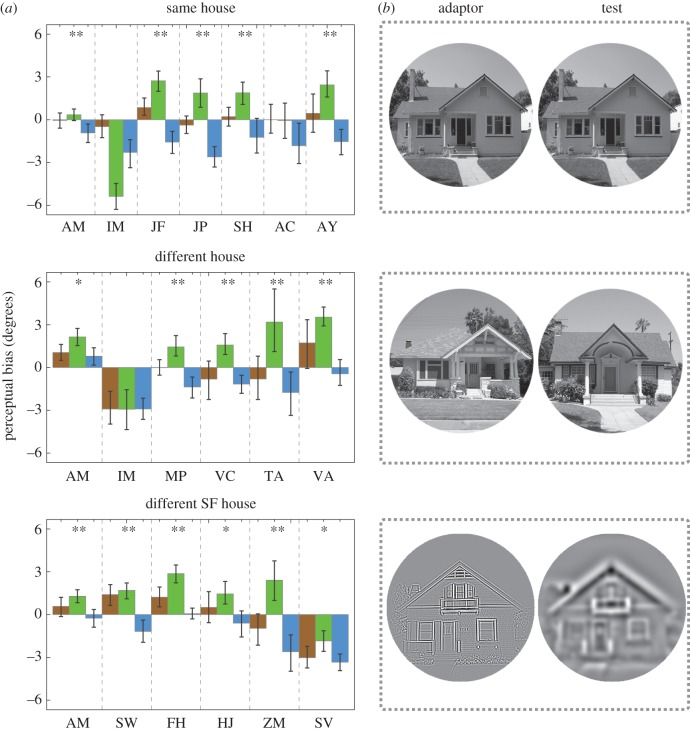
(*a*) Maximum-likelihood estimates of perceptual bias for baseline (brown), CW (green) and CCW (blue) blocks from the three conditions in Experiment 1. Error bars are bootstrapped 95% confidence intervals. Single asterisks (*) denote after-effects significant at the *α* = 0.05 level for repulsion. Double asterisks (**) denote after-effects also significant at the *α* = 0.001 level for repulsion. (*b*) Examples of adaptors and test stimuli used in each of the conditions tested (where necessary, contrast has been amplified for visibility).

In the *same house* condition, image H1 was used for both adaptor and test stimuli. In the *different house* condition, image H2 was the adaptor and image H3 was used for the tests (figure 1*b*). In the *different SF house* condition, the adaptor and test stimuli were images of the same house, but filtered to separate them for their SF content (figure 2*b*). In this condition, three different house images were used (H2, H4 and H5; figure 1*b*). Two observers were tested with H2, two with H4 and two with H5.

Lognormal filters were used for the *different SF house* condition. The filter used for adaptors had a peak SF of 10 cycles/degree. The filter used for the tests had a peak SF of 1.25 cycles/degree. Both filters had a full bandwidth at half-height of 1.5 octaves.

### 2.6. Methods specific to Experiment 2

**Figure 3.**
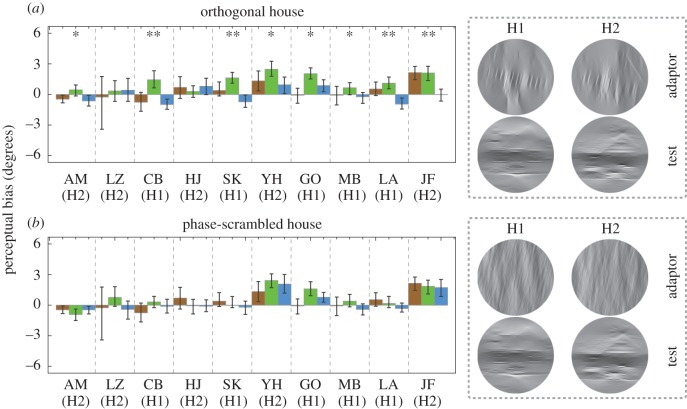
Maximum-likelihood estimates of perceptual bias for baseline (brown), CW (green) and CCW (blue) blocks from (*a*) the *orthogonal house* and (*b*) the *phase-scrambled house* conditions in Experiment 2. Error bars are bootstrapped 95% confidence intervals. Single asterisks (*) denote after-effects significant at the *α* = 0.05 level for repulsion. Double asterisks (**) denote after-effects also significant at the *α* = 0.001 level for repulsion. Examples of CW-tilted adaptors with untilted test stimuli used in each condition are illustrated to the right. The image number used for each observer is given below their initials.

All 10 observers participated in both the *orthogonal house* condition and the *phase-scrambled house* condition. In both conditions, adaptors were first tilted (either CW or CCW) and then filtered to retain Fourier energy close to vertical orientations (figure 3). Tests were upright images of the same house, initially filtered horizontally and then tilted by different amounts in each trial, as in Experiment 1. Five observers were tested using H1; the other five were tested using H2. For each observer, the adapting and test stimuli were differently filtered versions of the same house image. In the orientation domain, each filter was a Gaussian function of angle, centred on 0° (for the vertically filtered adaptors) or 90° (for the horizontally filtered tests); with a half-bandwidth at half-height of 23.5° and was clipped at ±40° from the peak, resulting in zero gain at orientations beyond the clip. In the *phase-scrambled* condition, tilted adaptors were phase-scrambled prior to orientation filtering, by adding a uniform distribution of random phase offsets (between –π and +π) to the Fourier phases of the image. The power spectra and RMS contrast of adaptors in the *phase-scrambled house* condition matched the power spectra and RMS contrast of adaptors in the *orthogonal house* condition. Identical (unscrambled), horizontally filtered, tilted tests were used in both conditions.

### 2.7. Psychophysical model

Data were analysed within the context of signal-detection theory, as described by Morgan *et al*. [23]. Within this model, the appearances of pedestal (*S*) and comparison (*C*) are normally distributed, i.e. *S* ∼ *N*(*p* + *µ*, *σ*^2^/2) and *C* ∼ *N*(*p* + *µ* + *t*, *σ*^2^/2), where *σ*^2^ is the variance of the performance-limiting noise, *p* is the pedestal tilt, *t* is the offset added to the comparison and *µ* is the perceptual bias specific to each test block. If there were no perceptual bias, then the distributions for pedestal and comparison would have means of *p* and *p* + *t*, respectively. The observer chooses the pedestal as closer to upright when it appears less tilted than the comparison. Accordingly, the probability of this choice *P*(^″^*S*^″^) = *P*(|*S*| < |*C*|) = *P*(*S*^2^/*C*^2^ < 1) has a doubly non-central *F* distribution. This distribution's denominator's non-centrality parameter is 2(*p* + *µ* + *t*)^2^/*σ*^2^, its numerator's non-centrality parameter is 2(*p* + *µ*)^2^/*σ*^2^, and both denominator and numerator have 1 d.f.

## 3. Results

From each block of trials (baseline, CCW and CW), we obtained maximum-likelihood estimates of bias *µ* and the variance of performance-limiting noise *σ*^2^. Negative biases with CCW adaptors and positive biases with CW adaptors are indicative of the repulsive TAE. Non-parametric bootstrapping (with bias-correction [24]) was used to quantify the reliability of our parameter estimates. The error bars shown in figures 2 and 3 contain the resultant 95% confidence intervals.

We also fit each observer's data from CCW-adaptor and CW-adaptor blocks simultaneously, forcing the bias parameter *µ* to be the same in both cases, but allowing *σ* to vary. The ratio *L*, between the likelihood of this nested model fit and the joint likelihood of the aforementioned separate fits to the same data is necessarily no greater than 1. To evaluate the ‘null’ hypothesis of no significant TAE in individual observers, we compare the criteria *α* = 0.05 and *α* = 0.001 to the value 1 – *F*(–2 ln *L*), where *F* is the cumulative *χ*^2^-distribution, with 1 d.f. This is known as the generalized likelihood-ratio test (see [25], pp. 440–441).

To evaluate null hypotheses at the group level, we performed one-sample *t*-tests using estimates of repulsion, which can be quantified either in degrees of tilt or in terms of the ‘just-noticeable difference’ (JND). A single value for repulsion, in degrees of tilt, can be obtained by subtracting one maximum-likelihood estimate of *µ* (the one obtained with CCW adaptors) from the complementary estimate (obtained with CW adaptors), and dividing the difference by 2. The ‘conspicuousness’ of repulsion can be quantified by further dividing this quotient by the JND. For the latter, we use the RMS of the maximum-likelihood estimates of *σ*. Results of the group-level *t*-tests appear in tables 1 and 2.

**Table 1.** Group-level statistics for repulsion in Experiments 1 and 2. *N* denotes the number of observers in each condition. The asterisk (*) denotes that the *p*-value was approaching significance. Removing observer IM from analysis makes the *p* = 0.002.

	repulsion (*R*)							
condition	*N*	mean *R* (°)	*t*-statistic (*R* > 0)	*p*-value	Cohen's *d*	paired *t*-statistic	*p*-value	Cohen's *d*
Experiment 1								
same house	7	1.13	2.25	0.066*	0.85			
different house	6	1.31	3.62	0.015	1.48			
different SF house	6	1.31	4.90	0.004	2.00			
Experiment 2								
orthogonal house	10	0.65	4.11	0.003	1.30	2.42	0.039	1.16
phase-scrambled house	10	0.20	2.68	0.025	0.85

**Table 2.** Group-level statistics for conspicuousness in Experiments 1 and 2. *N* denotes the number of observers in each condition. The asterisk (*) denotes that the *p*-value was approaching significance. Removing observer IM from analysis makes the *p* = 0.003.

	conspicuousness (*CI*)							
condition	*N*	mean *CI* (JND)	*t* –statistic (CI > 0)	*p*-value	Cohen's *d*	paired *t*-statistic	*p* -value	Cohen's *d*
Experiment 1								
same house	7	0.26	2.42	0.052*	0.91			
different house	6	0.27	4.24	0.008	1.73			
different SF house	6	0.33	5.84	0.002	2.38			
Experiment 2								
orthogonal house	10	0.21	4.36	0.002	1.38	2.88	0.018	1.30
phase-scrambled house	10	0.06	2.45	0.037	0.77			

### 3.1. Experiment 1

Estimates of bias (*µ*) from Experiment 1 are plotted in figure 2*a*. For the majority of observers, adaptation to a house tilted 15° (CCW of upright) produced a negative bias (relative to the baseline's bias) in subsequently viewed test houses, and adaptation to a house tilted –15° produced a positive bias. Generalized likelihood-ratio tests suggest after-effects significant at the *α* = 0.05 level for repulsion in the data from five of the seven observers in the *same house* condition, five of the six observers in the *different house* condition, and all six of the six observers in the *different SF house* condition. Group-level statistics appear in tables 1 and 2.

### 3.2. Experiment 2

Estimates of bias from Experiment 2 are plotted in figure 3. Generalized likelihood-ratio tests suggest after-effects significant at the *α* = 0.05 level for repulsion in the data from eight of the ten observers in the *orthogonal house* condition and none of the (same) 10 observers in the *phase-scrambled house* condition. Group-level statistics appear in tables 1 and 2. At the group level, both conditions produced mean repulsion and conspicuousness significantly larger than zero. However, a comparison using a paired-samples *t*-test between the means of the two conditions revealed that the *orthogonal house* condition produced a significantly larger repulsion compared with the *phase-scrambled house* condition (tables 1 and 2).

## 4. Discussion

Our results (Experiment 1) demonstrate that the TAE for natural scenes (houses) can be obtained with partially overlapping, yet different (and differently sized) adapting and test images, widely separated in SF content. Similar results have been obtained with sinusoidal gratings [18,26] and circular/radial patterns [19]. When after-effects survive manipulations of image, size and SF, their origin cannot be attributed to low-level visual mechanisms [22]. Our results extend Dekel & Sagi's [13] findings of TAEs with natural images as adaptors and sinusoidal gratings as tests, by showing that adaptation to global orientation can occur between adaptors and tests that are natural images. However, it is unclear from Experiment 1 whether the TAE for natural scenes arises because of interactions between high-level mechanisms selective for natural scenes, or whether it is simply a by-product of suppression between mid-level mechanisms, selective for spatial orientation in general.

To distinguish between these alternatives, in Experiment 2 we applied perpendicular filters to our stimuli, widely separating the orientation contents of adaptor and tests. Our finding of a repulsive TAE in this condition qualitatively differs from the assimilative ‘indirect effect’ found when retinally overlapping lines or gratings are separated between 60° and 87.5° [1]. We attribute this repulsion to our images' recognizability as slightly tilted scenes, rather than their Fourier image components. In support of this viewpoint, we found no after-effect at the individual observer level when the Fourier phases of our adaptors were scrambled. However, the group-level analyses did reveal a relatively small but significant TAE (tables 1 and 2), with phase-scrambled adaptors. This must be attributed to Fourier image components. A possible reason for this is that since man-made images are usually dominated by cardinal orientations, a sense of global tilt is still apparent in the images even after randomizing Fourier phase information (figure 3*b*, where randomized images might appear tilted CW).

Our most interesting finding is that vertically filtered houses induce repulsive TAEs. These TAEs were not only evident in most observers, but they were also much larger than the TAEs from phase-scrambled adaptors at the group level. Although our orientation-filtered houses are not as easily recognizable as their unfiltered counterparts, they possess clear higher-order structure, which is lacking in the phase-scrambled versions used for adaptation. Textures with similar higher-order (meaningless) structure are also more effective than phase-scrambled scenes as backward masks of ‘scene gist’ [27]. This suggests that textures with higher-order structure are fundamentally different from phase-randomized stimuli with similar orientation statistics. Nonetheless, the after-effect of adapting to tilted buildings is different from the after-effect elicited by the perception of a global form contained in meaningless textures. Although our Experiment 2 showed that the former can survive large differences between the orientation contents of adaptor and test, the latter cannot [19].

Our results are unique in the literature on the appearance of uprightness, because they show that the global orientation of a scene can be encoded separately from its local feature content. It is assumed that information about scene orientation is embedded in the early global percept of scene layout, a property which is rapidly extracted when looking at a scene [17,28]. Based on this assumption, at present, we can only speculate regarding where selectivity for the orientation of natural scenes arises in the brain. One possible candidate is the parahippocampal place area, which is thought to encode scene layout rather than object content [29]. In support of this, such scene selective regions are known to be responding similarly to scenes containing only close-to-vertical or close-to-horizontal orientations [30], akin to the stimuli we used here. Different local feature content can therefore lead to the encoding of similar global spatial layout in scenes, which presumably is what led to a repulsive TAE from vertically filtered adaptors on horizontally filtered tests.

As noted in the Introduction, the TAE is routinely invoked as a manifestation of the mutual inhibition between visual mechanisms selective for orientation. Consequently, the natural conclusion to draw from our results is that there must be mechanisms selective for the orientations of images with meaningful, higher-order structure. Of course, we cannot say whether those mechanisms are mutually inhibitory, or whether the TAE for natural scenes should be attributed to their modulation of lower level mechanisms. Indeed, other authors have invoked pre-saccadic remapping in space [18], top-down modulation of low-level feature detectors through feedback from form processing regions [19] and selective attention [26] in attempts to explain how the TAE can survive the spatial separation of adaptor and tests.

One further possibility is normalization. Extensive real-world experience with close-to-upright scenes (canonical orientation) may have resulted in the establishment of uprightness as a norm against which other orientations are compared. Exposure to tilted scenes may simply shift the subjective norm of uprightness towards the tilted direction, which then results in an objectively upright scene seen as tilted away. Indeed, Asch & Witkin [15] report that tilted scenes eventually appear upright over extended viewing, implying normalizing towards uprightness.

## Data accessibility

Raw behavioural data from both experiments can be accessed at the Dryad Digital Repository: http://dx.doi.org/10.5061/dryad.6pv38 [31].
